# Risk of malignancy in thyroid nodules: predictive value of puncture feeling of grittiness in the process of fine-needle aspiration

**DOI:** 10.1038/s41598-017-13391-3

**Published:** 2017-10-12

**Authors:** Jieli Luo, Chao Zhang, Fengbo Huang, Jianshe Chen, Yang Sun, Kailun Xu, Pintong Huang

**Affiliations:** 1 Department of Ultrasound, the Second Affiliated Hospital of Zhejiang University School of Medicine, Zhejiang, 310009 China; 2 Department of Pathology, the Second Affiliated Hospital of Zhejiang University School of Medicine, Zhejiang, 310009 China

## Abstract

Fine-needle aspiration cytology (FNAC) is widely used for diagnosing thyroid nodules. However, there has been no specific investigation about the puncture feeling of grittiness. The aim of the present study was to see if the puncture feeling of grittiness during fine-needle aspiration procedure, combined with standard FNAC, could improve the accuracy in diagnosing thyroid cancer. A total of one thousand five hundred and thirty-one thyroid FNAC specimens acquired between January 2013 and January 2017 were retrospectively retrieved. All cases underwent surgical intervention. The FNAC diagnoses and puncture feeling of grittiness were evaluated and compared with the results of final histopathological diagnoses. The sensitivity, specificity, positive predictive value (PPV) and negative predictive value (NPV) of diagnosis for thyroid nodules by FNAC alone, puncture feeling of grittiness alone, and the combination of FNAC plus grittiness were calculated respectively. The findings of our study suggest that puncture feeling of grittiness is a useful adjunct. Adding puncture feeling of grittiness to FNAC can significantly enhance the ability to differentiate malignant thyroid nodules from benign thyroid nodules. More importantly, we found that puncture feeling of grittiness is surprising trust-worthy in being near perfectly reproducible per individual radiologist, and among different operators.

## Introduction

The incidence of thyroid carcinoma is increasing in recent years, accounting for 10%–15% of thyroid nodules^1–3^. Fine needle aspiration cytology (FNAC) plays an important role in the thyroid nodules by estimating the risk of malignancy. The main purpose of FNAC is to prevent unnecessary surgeries for benign conditions and to avoid missing malignant nodules^4–6^. With the improvement and implementation of the Bethesda system for reporting thyroid cytopathology (TBSRTC), FNAC is proved to be a reliable and cost-effective method for the diagnosis of thyroid nodules. It can help decide whether thyroid nodules should be managed expectantly or surgically^7–10^.

However, FNAC is not a perfect gold standard for diagnosing thyroid nodule with overall false negative rates (malignant histology of a nodule with benign cytology) ranging from <1% to 12%^11–13^. Also, thyroid nodules with indeterminate cytological findings still remain a matter of debate^14^. Sometimes it is difficult to say whether a patient should be submitted to surgery or not. In order to improve diagnostic sensitivity and accuracy, our study explored the utility of the puncture feeling of thyroid nodules during fine-needle aspiration (FNA) procedure. To the best of our knowledge, there were no literature reporting on the role of puncture feeling of grittiness during FNA procedure about thyroid nodules.

One can get various feelings in the process of FNA, such as needle getting stuck, hard to pull and so on. Dayan et.al reported that at the base of FNA needle equipped with a piezoelectric force-sensor could precisely measure the forces opposing needle penetration quantitatively, but it tested on the phantom which was composed of gelatin and needed special equipment^15^. Grittiness is a heretofore well defined feeling when the tip of needle meets special structures, for example, a psammoma body. No relative research has been reported about the association between the puncture feeling of grittiness and FNAC pathology. The purpose of the present study, therefore, was to investigate the benefits of puncture feeling of grittiness, in addition to FNAC, for the diagnosis of thyroid nodules. Further, we investigated the repeatability of puncture feeling.

## Results

**Figure 1:**
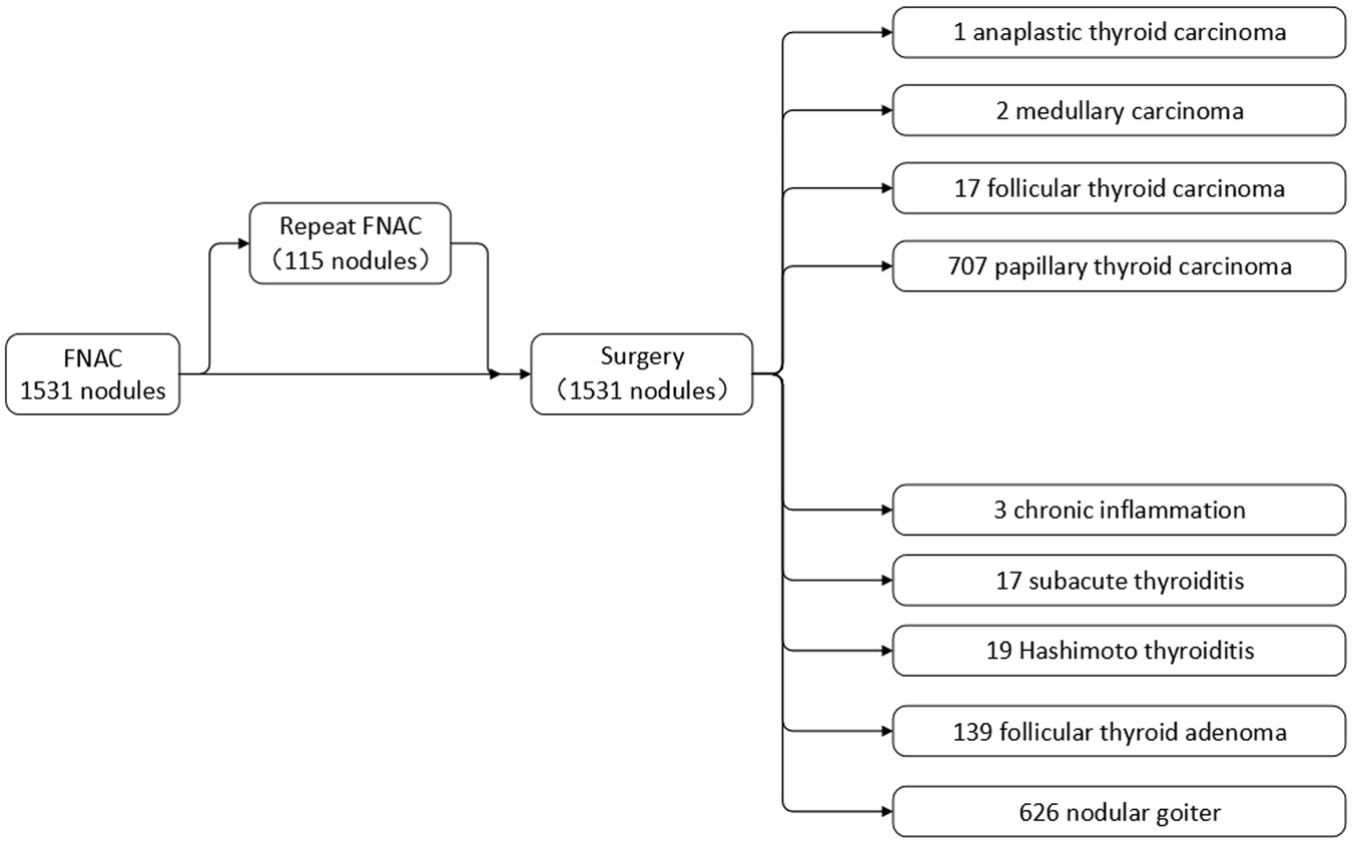
Flow diagram of the thyroid nodule selection process.

From January 2013 and January 2017, a total of 1531 thyroid nodules that underwent US guided FNA in our department of ultrasound were submitted to the thyroid surgery. Of those, there were 414 (27.04%) males and 1117(72.86%) females, a male to female gender ratio of 1:2.7. The mean age was 45.5 ± 9.7 (range 19–82) years in this cohort. Seven hundred and twenty-seven thyroid nodules were diagnosed as thyroid malignancies on final histopathology. Among them, 707 were papillary thyroid carcinomas (PTC), 17 were follicular thyroid carcinomas, 2 were medullary carcinomas and 1 was anaplastic thyroid carcinoma. Eight hundred and four lesions were benign: nodular goiter in 626 cases (77.86%), follicular thyroid adenoma in 139 cases (17.29%), Hashimoto thyroiditis in 19 cases (2.36%), subacute thyroiditis in 17 cases (2.11%) and chronic inflammation in 3 cases (0.37%). The pathological classification of all 1531 thyroid nodules is shown in Fig. 1.

One thousand five hundred and thirty one thyroid nodules were classified into six categories on the basis of the FNAC results according to the Bethesda System as follows: there were 75(4.90%) unsatisfactory or non-diagnostic nodules, 324(21.16%) benign nodules, 181 (11.82%) atypia of undetermined significance/follicular lesion of undetermined significance (AUS/FLUS) nodules, 236(15.41%) follicular neoplasm/suspicious for a follicular neoplasm (FN/SFN) nodules, 128 (8.36%) suspicious for malignancy (SM) nodules and 587(38.34%) malignant nodules. Among 1531 thyroid nodules, 726 nodules had puncture feeling of grittiness at first FNAC, 86 out of 726 were benign, the remaining 640 nodules were malignant. The false positive rate of puncture feeling of grittiness was 11.85%. Meanwhile, as for 805 nodules with absence of puncture feeling of grittiness, 87 nodules were malignant and 718 nodules were benign. The false negative rate of puncture feeling of grittiness was 10.81%. Furthermore, 49 nodules had puncture feeling of grittiness and 767 nodules did not yield puncture feeling of grittiness at Bethesda Categories I–IV. For the 49 nodules with puncture feeling of grittiness in the Categories I–IV, 44 nodules were PTC, 1 nodule was chronic inflammation, 4 nodules were Hashimoto thyroiditis. Six hundred and seventy- seven nodules had puncture feeling of grittiness and 38 nodules did not have puncture feeling of grittiness at Bethesda Categories V and VI. For the 38 nodules without puncture feeling of grittiness in the Categories V–VI, 12 nodules were follicular thyroid carcinomas, 24 nodules were PTC, 2 nodules were follicular thyroid adenomas. (Table 1).

**Table 1:** The distribution of FNAC and puncture feeling results, compared with pathology.

FNAC Pathology	I–IV		V–VI	
	with grittiness	without grittiness	with grittiness	without grittiness
Malignant	44	51	596	36
Benign	5	716	81	2

A repeat FNAC was performed in 67 out of 75 patients with unsatisfactory or non-diagnostic cytology, 12 out of 324 patients with benign cytology, 5 out of 181 patients with AUS/FLUS cytology, 4 out of 236 patients with FN/SFN cytology and 27 out of 128 patients with SM cytology. At Bethesda Categories I–IV, 11 nodules had puncture feeling of grittiness and 77 nodules did not have puncture feeling of grittiness at repeat FNAC, 14 nodules had puncture feeling of grittiness and 74 nodules did not have puncture feeling of grittiness at initial FNAC. At Bethesda Categories V and VI, twenty-six nodules had puncture feeling of grittiness and 1 nodule did not have puncture feeling of grittiness at repeat FNAC, 26 nodules had puncture feeling of grittiness and 1 nodule did not have puncture feeling of grittiness at initial FNAC. The inter-observer and intra-observer agreement on whether grittiness was feel when puncturing thyroid nodules was shown in Tables 2, 3. The inter-observers and intra-observers consistency analysis revealed that the agreement on puncture feeling of grittiness were almost perfect with k of 0.842 (95%, CI: 0.676–0.999, *P* < 0.05) and k of 0.881 (95%, CI: 0.712–1.000, *P* < 0.05) respectively.

**Table 2:** Assessment of intra-observer variability of puncture feeling of grittiness in the process of fine-needle aspiration.

Initial FNAC	Repeat FNAC	
	with grittiness	without grittiness
with grittiness	16	2
without grittiness	1	43

**Table 3:** Assessment of inter-observer variability of puncture feeling of grittiness in the process of fine-needle aspiration.

Radiologist A	Radiologist B	
	with grittiness	without grittiness
with grittiness	19	3
without grittiness	1	30

The sensitivity, specificity, accuracy, positive predictive value (PPV) and negative predictive value (NPV) of single FNAC were 86.93%, 89.68%, 88.37%, 88.39%, 88.36%. That for puncture feeling of grittiness were 88.03%, 89.30%, 88.70%, 88.15%, 89.19%, respectively. After FNAC and puncture feeling of grittiness data were combined, the sensitivity, specificity, accuracy, PPV and NPV were 92.98%, 89.05%, 90.92%, 88.48%, 93.35%, respectively. The combined data compared with either FNAC alone or puncture feeling of grittiness alone, the sensitivity, accuracy, NPV were statistically significant (*P* = 0.000, 0.001; *P* = 0.021, 0.042 and *P* = 0.001, 0.004). Table 4 lists the sensitivity, specificity, PPV, NPV, and diagnostic accuracy for FNAC, puncture feeling of grittiness and combined FNAC and puncture feeling of grittiness in detail.

**Table 4:** Diagnostic performance of FNAC, puncture feeling of grittiness and combined FNAC and puncture feeling of grittiness.

Examination method		Pathology		Accuracy	Sensitivity	Specificity	NPV	PPV
		Malignant	Benign					
FNAC	malignant	632	83	88.37%	86.93%	89.68%	88.36%	88.39%
	benign	95	721					
Grittiness	malignant	640	86	88.70%	88.03%	89.30%	89.19%	88.15%
	benign	87	718					
combined FNAC and Grittiness	malignant	676	88	90.92%	92.98%	89.05%	93.35%	88.48%
	benign	51	716					

Note: NPV, negative predictive value; PPV, positive predictive value.

**Figure 2:**
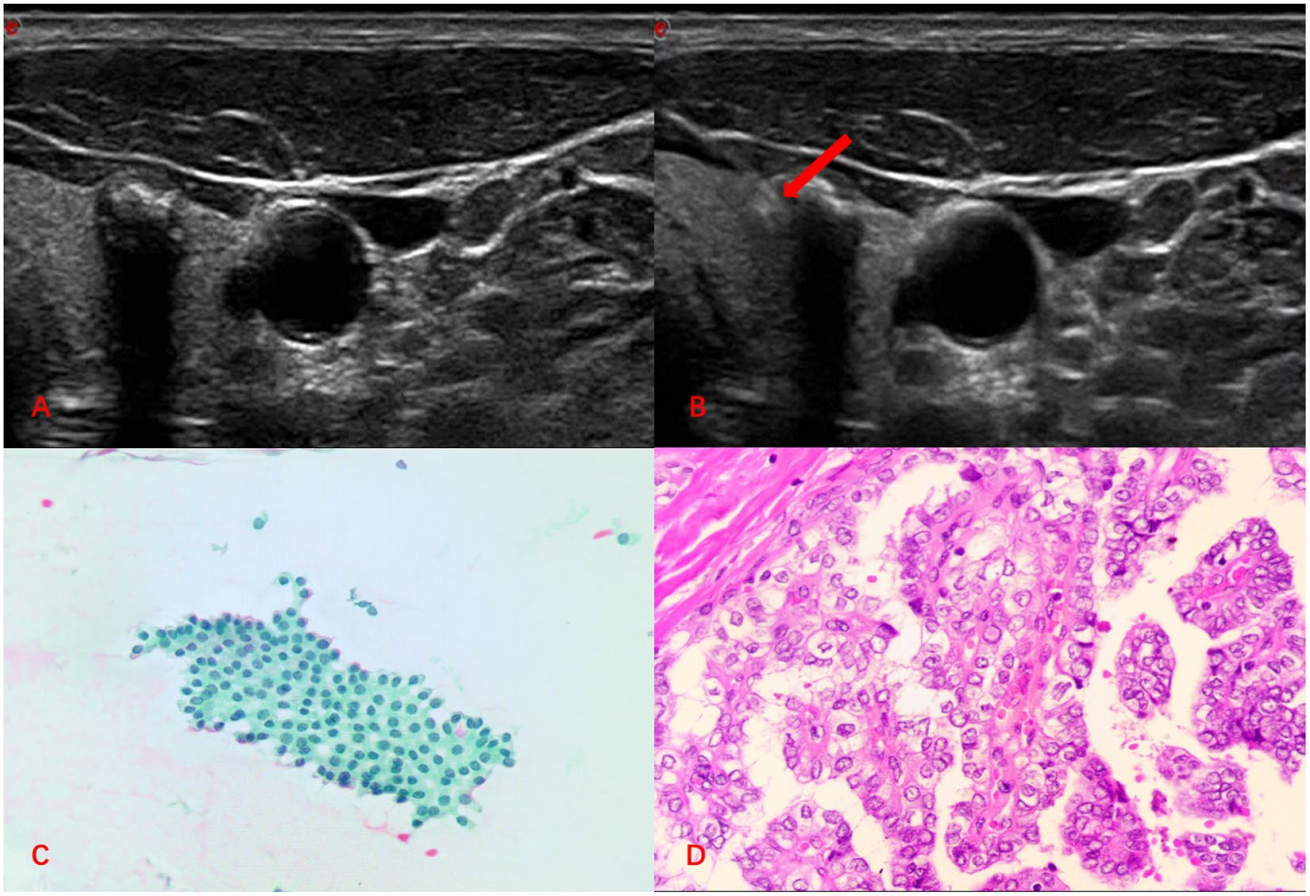
Image of a 46-year-old man with a thyroid nodule in the right lobe. (**A**) The two-dimensional ultrasound showed a lesion with microcalcification in the right lobe. (**B**) On FNA, the needle tip could penetrate through the calcified nodule (Red arrow). (**C**) FNAC smear showing features of a benign thyroid nodule (Papanicolaou, ×400). (**D**) Histopathology showing features of papillary carcinoma (Hematoxylin and eosin, ×400).

**Figure 3:**
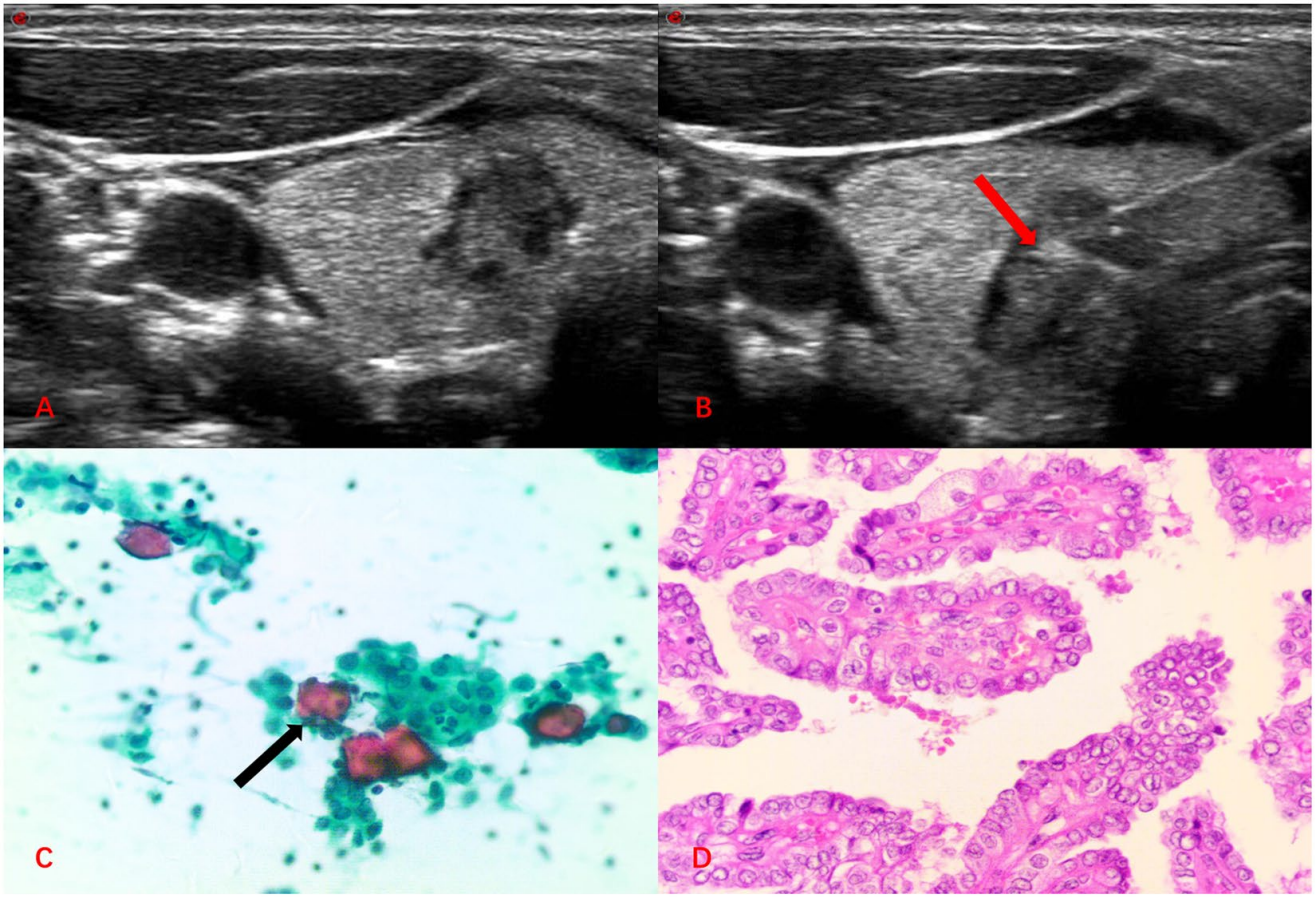
Image of a 39-year-old woman with a thyroid nodule in the left lobe. (**A**) The two-dimensional ultrasound showed a lesion with irregular margins in the left lobe. (**B**) On FNA, the needle tip passed through the center of the nodule (Red arrow). (**C**) FNAC smear showing features of malignant thyroid nodule, for example, pasmmoma body (Black arrow, Papanicolaou, × 400). (**D**) Histopathology showing features of papillary carcinoma (Hematoxylin and eosin, × 400).

No major or minor complications happened in the process of FNAC in our study. We present two cases for illustration. First case, a malignant thyroid nodule with a false negative FNAC cytological reading, having feelings of grittiness in the process of puncture (Fig. 2). Second case, a malignant thyroid nodule with a malignant FNAC cytological result (nuclear crowding and overlapping or enlargement, poorly formed inclusions, pasmmoma bodies and so on), having grittiness in the process of puncture, a pasmmoma body is shown in section (Fig. 3).

## Discussion

Recent studies have reported an increasing trend in the incidence of thyroid cancer^16^. PTC is the most common type of all types of thyroid carcinomas, accounting for 80% to 85% of all^17–19^. Obviously, it is important to differentiate benign and malignant thyroid nodules. FNAC performed under ultrasound guidance has been reported to be the most sensitive and reliable tool for the detection of thyroid malignancy and selecting candidates for surgery. But preoperative FNAC cannot detect all malignant thyroid nodules (false negative), especially in cases of hypo-cellular lesions such as deep nodules with cystic changes^20^. Our study revealed for the first time puncture feeling in the process of FNAC contributes to improving this situation.

It had been reported that a robot could reliably detect the instant of needle puncture using coaxial force sensor without being affected by artifacts^21^. In reality, it is also easy to sense the tissue properties using free hand for skilled radiologists, such as the skin, the muscle, the ligament, etc^22–24^. Piercing tip through the thyroid nodules could yield feeling of mainly sticky, soft with little resistance, mushy and so on. However, there have been no studies specifically regarding the significance of the feeling of grittiness. Our study formally investigated puncture feeling of grittiness and whether it was associated with malignant thyroid nodule. The presence of grittiness was significantly correlated with higher incidences of malignant thyroid nodules (*P* < 0.05), compared with absence of grittiness in our study. Thus, our results suggested that puncture feeling of grittiness was associated with malignant lesions.

The origin of the puncture feeling of grittiness might be calcifications. Ultrasonographically detected calcifications can be classified as macro- or micro-calcification^25^. The most common form of intra-nodular calcification observed by thyroid ultrasound is the dense and peripheral type. Nodular micro-calcification is the most useful indicator for the diagnosis of malignant thyroid nodule^26^. Cytological and histological examination demonstrated that psammoma bodies mostly correspond to micro-calcification. Psammoma bodies are defined as spherical calcified foci with concentric laminations, and is different from intra-follicular inspissated colloid. There are usually situated within stromal stalks of tumor papillae^27–29^. Other calcifications are characterized by a spherical shape lacking laminations or by an irregular shape with laminations. Although psammoma bodies are found in up to 50% of papillary thyroid carcinomas, their clinicopathological significance remains uncertain^30^. The diagnostic characteristic of papillary thyroid carcinoma is the presence of psammoma bodies in pathology^31^. Psammoma bodies in thyroid nodule may be formed by calcification and necrosis of intravascular or intra-lymphatic tumor thrombi. Others claimed that psammoma bodies were regarded as intracellular calcifications in viable cells of the nidus^32^. The definite mechanisms responsible for the formation of psammoma bodies remain unknown.

The findings of the present study suggested that the presence of psammoma bodies under the microscope may be a useful prognostic indicator of malignancy, although psammoma bodies are occasionally observed in benign thyroid nodule as well. 44 of 95 false negative cases found on FNAC in the study could have been correctly diagnosed as carcinoma by adding puncture feeling of grittiness. These results suggested that the presence of grittiness might be a useful predictor. Though puncture feeling of grittiness appeared to suggest a higher incidence of malignancy than those with no grittiness (*P* < 0.05). Among 36 malignant thyroid nodules without puncture feeling of grittiness, 12 nodules were follicular thyroid carcinomas, 24 nodules were PTC. The false negative rate of puncture feeling of grittiness in predicting malignant thyroid nodules was 10.81% in this study. One of the main reasons for false negative of puncture feeling of grittiness might be that psammoma bodies were present in up to 50% of papillary thyroid carcinomas and were never present within follicular thyroid carcinomas. Contrary, the main reason for false positive results in puncture feeling of grittiness in this study may due to the benign thyroid nodules with severe fibrosis, hyalinization, dense calcification, hemorrhage and necrosis. Overall, our results suggested that puncture feeling of grittiness together with FNAC would be a useful decision-making tool particularly for suspicious thyroid nodules.

Some investigators have suggested that repeat FNAC should be performed in order to reduce the risk of false negative results. Factors that lower the probability of aspirate representativeness include small size of nodule, mixed cystic-solid character of the nodule and difficult localization^33–35^. Others suggest that repeat FNAC is not reasonable as they rarely change the category of Bethesda classification while exposing patients to stress and additional costs^11,36,37^. According to the 2015 American Thyroid Association’s guidelines^38^, repeat FNAC should be done with ultrasound guidance for the thyroid nodule yielding an initial non-diagnostic cytology result. A repeat FNAC could lead to a more definitive cytologic diagnosis in many cases. Repeat FNAC is the usual method of management after indeterminate diagnosis because the expected risk of malignancy is far more than 5% according to Koh J *et al*.’s study^39^. Repeat FNAC appears to not increase the expected likelihood of a malignant finding for patients who have an initial benign reading according to Graciano AJ *et al*.’s research^40^.

In our study, 115 thyroid nodules underwent repeat FNAC. The inter-observers and intra-observers consistency analysis showed that the agreement in puncture feeling of grittiness were almost perfect with k of 0.842 (95% CI: 0.676–0.999, *P* < 0.05) and k of 0.881 (95% CI: 0.712–1.000, *P* < 0.05) respectively. This result showed that puncture feeling of grittiness had a high repeatability rate whether for the same radiologist or different radiologists. Although the puncture feeling of grittiness was a very subjective feeling, the agreement of subjective puncture feeling for one radiologist and for different radiologists was surprisingly good. One reason behind this could be that, ultrasound-guided puncture was performed on the most suspicious spot based on ultrasound images whether by the same or by different radiologists. Another reason was that puncture feeling of grittiness seems very obvious for a skilled radiologist.

This study has a number of limitations. Firstly, it employed a retrospective design involving a single medical center. As such, selection bias might have been present. Some patients were excluded for not needing surgeries. Some patients with benign cytological results may not have been enrolled to our research, resulting in a higher incidence of malignant nodules. Secondly, thyroid nodules with more suspicious characters at ultrasound and other risk factors are more likely to undergo FNAC and surgery. Patients with multiple nodules is another potential source of selection bias. Furthermore, the difference in the reliability of puncture feeling of grittiness between papillary and follicular lesions had not been compared due to the insufficiency of follicular thyroid lesions samples. In addition, this study did not specially address ultrasonic appearance, such as taller than wider shape, echogenicity and so on, in making clinical decisions. Although puncture feeling of grittiness has a high repetition reliability, it is still a subjective sensation. Only well experienced radiologists can develop this feel. The puncture feeling of grittiness in this research is confined to veteran radiologists. What’s more, the puncture feeling comes from the force at the needle tip as well as the friction to the needle sidewall. When the needle is inserted to greater depth, friction dominates and dilutes the feel from the needle tip. Finally, the puncture feeling corresponding to histology had not been confirmed pathologically in this study, which is one of the subjects of ongoing research in the future.

It was apparent that the combined application of FNAC and puncture feeling of grittiness contributed to higher sensitivity, accuracy, NPV and PPV than FNAC alone or puncture feeling of grittiness alone in the study. In conclusion, FNAC/puncture feeling of grittiness reduces inconclusive diagnoses and increases the sensitivity compared to FNAC without paying attention to the feeling of grittiness. This expedites surgery when indicate. The findings of the present study suggest that puncture feeling of grittiness combined with FNAC is a useful method to differentiate benign and malignant thyroid nodules. Moreover, the reproducibility of puncture feeling of grittiness is surprising high.

## Methods

This retrospective study was approved by Institutional Review Board and local ethics committee of the Second Affiliated Hospital of Zhejiang University School of Medicine. Written standard informed consent was obtained from each patient prior to FNAC. The methods in this study were performed in accordance with approved guidelines. No incentives, financial or other, were offered to them.

### Patients

One thousand five hundred and thirty-one thyroid nodule FNACs performed at the department of ultrasound, the Second Affiliated Hospital of Zhejiang University School of Medicine, from January 2013 to January 2017 were retrieved retrospectively. FNAC was performed under ultrasound guidance. Patients had to be excluded from the analysis due to multiply nodules on one side of thyroid or without FNAC or surgery results (Fig. 1). Medical records of thyroid nodules were subsequently reviewed using a picture archive and communications system (PACS). Clinicopathological parameters such as gender, age, ultrasonographic characters and pathologic examinations were evaluated by reviewing clinical charts and pathological records. All patients in this cohort underwent thyroid ultrasonography, preoperative FNAC diagnoses and postoperative histologic diagnoses.

### Ultrasonography and fine-needle aspiration

Radiologists with expertise in routine ultrasound examinations performed thyroid ultrasonography using an 8~14 MHz linear transducer (Mylab 90, Esaote Medical System, Italy). Thyroid nodule diagnostic FNAC was recommended for: (a) cases with one or more suspicious sonographic features: microcalcifications, extreme hypoechogenicity (lower than cervical strap muscle), taller than wide shape, irregular margins (infiltrative, speculated, or microlobulated), rim calcifications with small extrusive hypoechoic soft tissue component and extrathyroidal extension. (b) Metastatic cervical lymph nodes. (c) Distant metastases. (d) History of cervical irradiation. (e) Per patient request despite advice to the contrary. (f) Fear of turning malignant. (g) Compression and uncomfortable symptoms. Ultrasonographic features were being used to select the most suspicious thyroid nodule for FNAC in patients with multiple thyroid nodules.

All ultrasound guided FNACs were performed by radiologists with at least 5 years of dedicated experience in performing thyroid ultrasound. The patient’s neck was supported by a pillow with the neck exposed and the head extended. After administration of subcutaneous and perithyroidal local anesthesia with 1% lidocaine, each nodule underwent capillary aspiration with the freehand technique. At least four needle passes were made in order to obtain adequate specimens with 22-gauge needles (Hakko, Japan). Puncture time depended on the pathologist and the quality of slides. If the specimen contains at least six groups of benign follicular cells with each group being composed of at least ten cells, the specimen was considered satisfactory. The smears were immediately fixed in 95% alcohol for Papanicolaou staining. At the same time, an assistant recorded whether the nodule had puncture feeling of grittiness or not according to radiologist. Real-time ultrasound was used to monitor all procedures continuously in order to assess complications such as parenchymal edema or perithyroidal hematoma. Each biopsy site was monitored under local compression for at least 30 minutes.

One hundred and fifteen thyroid nodules underwent repeat FNAC no sooner than three months after initial FNAC, for these conditions: (1) Some nodules with an initial non-diagnostic cytology result. (2) Ultrasound and clinical characteristics predictive for malignancy despite benign cytological results. (3) Patients strongly wishing a repeat FNAC. A radiologist with at least 5 years of experience blinded to the cytological result and puncture feeling would perform the repeat FNAC. Likewise, a pathological with a minimum of 5 years of experience blinded to evaluation of repeat FNAC cytological specimens was involved. The Kappa coefficient was used to determine inter-observer and intra-observer reliability of puncture feeling of grittiness at first FNAC and on repeat FNAC.

The National Cancer Institute (NCI) proposed a six tier system named TBSRTC has been adopted in our institution since 2011^41^. Cytological diagnoses were classified as follows: Bethesda I was unsatisfactory or non-diagnostic; Bethesda II was benign; Bethesda III was AUS/FLUS; Bethesda IV was FN/SFN; Bethesda V was SM; Bethesda VI was malignant. According to the Bethesda System, <grade V were benign and Bethesda Category V and VI were considered malignant.

### Surgery

Surgery was considered for patients with: (1) Cytologically non-diagnostic nodule with highly suspicious ultrasonographic features, growth of nodule (>20% in two dimensions) during follow-up, or other clinical risk factors; (2) FNA Bethesda Category ≥ III; (3) Neck discomfort; (4) Dysphagia; (5) Cosmetic problem. (6) Per patient request despite advice to the contrary. All sections stained with hematoxylin and eosin from surgery were screened by pathologists with at least 5 years of specific experience.

### Statistical analysis

Data analysis was performed with SPSS software for Windows v19.0 (IBM, USA). The level of statistical significance was defined as *P* < 0.05 with a confidence interval of 95%. Concordance between the categorical data obtained by two same or different radiologists was evaluated using the kappa analysis. The sensitivity, specificity, negative predictive value, positive predictive value, and accuracy of diagnosis of thyroid were calculated for puncture feeling of grittiness and FNAC based on the final histological results. Comparison of Categorical variables were evaluated using the chi square analysis.
